# NBS1 plays a synergistic role with telomerase in the maintenance of telomeres in *Arabidopsis thaliana*

**DOI:** 10.1186/1471-2229-12-167

**Published:** 2012-09-17

**Authors:** Lucie Najdekrova, Jiri Siroky

**Affiliations:** 1 Department of Plant Developmental Genetics, Institute of Biophysics of the Czech Academy of Sciences, Kralovopolska 135, Brno, 61265, Czech Republic

## Abstract

**Background:**

Telomeres, as elaborate nucleo-protein complexes, ensure chromosomal stability. When impaired, the ends of linear chromosomes can be recognised by cellular repair mechanisms as double-strand DNA breaks and can be healed by non-homologous-end-joining activities to produce dicentric chromosomes. During cell divisions, particularly during anaphase, dicentrics can break, thus producing naked chromosome tips susceptible to additional unwanted chromosome fusion. Many telomere-building protein complexes are associated with telomeres to ensure their proper capping function. It has been found however, that a number of repair complexes also contribute to telomere stability.

**Results:**

We used *Arabidopsis thaliana* to study the possible functions of the DNA repair subunit, NBS1, in telomere homeostasis using knockout *nbs1* mutants. The results showed that although NBS1-deficient plants were viable, lacked any sign of developmental aberration and produced fertile seeds through many generations upon self-fertilisation, plants also missing the functional telomerase (double mutants), rapidly, within three generations, displayed severe developmental defects. Cytogenetic inspection of cycling somatic cells revealed a very early onset of massive genome instability. Molecular methods used for examining the length of telomeres in double homozygous mutants detected much faster telomere shortening than in plants deficient in telomerase gene alone.

**Conclusions:**

Our findings suggest that NBS1 acts in concert with telomerase and plays a profound role in plant telomere renewal.

## Background

The ends of linear eukaryotic chromosomes are protected by specific chromatin structures called telomeres that are composed of tandemly repeated telomeric DNA and proteins. In vertebrates, six specific proteins associate with telomeres having affinity to either single-stranded or double-stranded telomeric DNA and they are collectively called shelterin [1]. These complex structures are essential for chromosome stability, as they differentiate chromosome ends from DNA double-strand breaks (DSBs) [2]. They protect chromosome termini from nucleolytic attack and undesirable recombination. Telomeres also counterbalance incomplete replication of terminal DNA by conventional DNA polymerase [3]; cells have evolved specific telomerase reverse transcriptase (TERT), which can synthesise telomeric repeats using its own RNA template thus ensuring proper telomere length. In general, eukaryotic telomeres are composed of tandem G/C rich repeats that end in a single strand 3' overhang which can fold back and invade the duplex repeats to form the so-called T-loop [4]. In the absence of telomerase, telomeres become non-functional, shorten with successive cell divisions, and chromosome termini can fuse as a consequence of de-protection. Their fusion is a result of the non-homologous-end-joining (NHEJ) which is the prevailing mechanism of DSB healing in plants. Although numerous attempts have been made to assess shelterin counterparts in plants, to date these proteins have not been identified (see [5] for review). Together with proteins invariantly occurring at the telomeres and providing a telomere "capping" function, many additional protein complexes are regularly found in eukaryotic telomeres. Paradoxically, many of these proteins are involved in DNA repair or recombination and this seems inappropriate at the telomere ends which should be hidden from recombination events and end-to-end fusions.

Over the past decade, an increasing body of data has accumulated on the concerted network of DNA repair factors and various protein kinases following the disruption of DNA integrity, including noxious extrinsic factors, DNA replication errors, checkpoint signalling and, meiotic and somatic recombination. One system that plays an essential role in DNA repair, recombination, DNA replication as well as in the cell cycle checkpoint activation and telomere maintenance, is the MRN complex (for recent reviews see [6,7]). This consists of three subunits (MRE11-RAD50-NBS1). A single NBS1 molecule is associated with two dimers of MRE11 and RAD50. The MRE11 and RAD50 proteins form a heterotetramer which contains two DNA-binding and processing domains that can bridge free DNA ends [8]. In *Saccharomyces cerevisiae*, this complex comprises subunits MRE11, RAD50, and XRS2. Whereas both proteins, MRE11 and RAD50, share a high homology across various eukaryotes, the XRS2 protein has a lower degree of homology with NBS1, which is specific for mammals and plants [9], although a functional homologue of NBS1 has been found in *Schizosaccharomyses pombe*[10].

In humans, mutation in the *NBS1* gene leads to the chromosomal instability disorder, Nijmegen breakage syndrome 1. Besides other clinical hallmarks, this syndrome is associated with enhanced sensitivity to ionizing radiation and chromosomal instability which leads to early developing cancer even in *NBS1*^+/−^ heterozygotes [11]. Murine *NBS1*^+/−^ heterozygotes are phenotypically normal although complete removal of the NBS1 is embryonically lethal in mice [12]. Accumulating evidence demonstrates that NBS1 interacts with telomeres and contributes to their stability, at least in human and mouse cells (reviewed in [11]). Direct interaction of NBS1with telomere repeat-binding factor 1, TRF1, has been shown for immortalized telomerase negative cells [13] implying that this interaction might be involved in the alternative lengthening of telomeres. Moreover, it has been shown by indirect immuno-fluorescence that NBS1 co-localise with a shelterin constituent, telomere repeat-binding factor 2 (TRF2), during the S phase in cultured HeLa cells [14], possibly by modulating t-loop formation. Similarly, in mouse embryonal fibroblasts, active recruitment of NBS1 to dysfunctional telomeres has been observed [15].

It is known that MRE11 and RAD50 together with protein kinases ATM and ATR, are also essential for proper telomere maintenance in plants (Reviewed in [5,16,17]). Inactivation of *Arabidopsis RAD50* or *MRE11* leads to hypersensitivity to ionizing radiation or radiomimetics and reduced plant health and even sterility. In knockout *RAD50* or *MRE11* mutant *Arabidopsis* plants genome instabilities are induced, including chromosome end-to-end fusions [18-21]. Absence of RAD50 led to rapid shortening of telomeres and loss of telomere repeats accompanied by chromosome-end fusions, while in double mutant plants (*rad50/tert*) a synergistic effects of RAD50 and telomerase on the frequency of bridges have been found [19], demonstrating the dual role of the RAD50 protein in plants. A homolog of the third MRN constituent, NBS1, has been isolated in the higher plants, *Arabidopsis thaliana* and *Oryza sativa*[22]. The NBS1 proteins from both plant species were shown to be smaller in size than animal NBS1, but both contained typical domains such as the FHA (forkhead-associated), BRCT (BRCA1 C Terminus) domain, the MRE11-binding domain, and the ATM-interacting domain. Functional analysis using yeast two-hybrid assay showed that the *Os*NBS1 protein interacted not only with plant MRE11 but also with animal MRE11. *OsNBS1* mRNA expression was found to be higher in the shoot apex and young flower and *AtNBS1* expression increased when plants were exposed to X-rays [22]. Cytogenetic analyses showed numerous anomalies including the fragmentation of meiotic chromosomes in At*nbs1* knockout mutants [23], although on simultaneous inactivation of plant ATM.

In this study, we examined the role of NBS1 in telomere maintenance in the plant model species *Arabidopsis thaliana*. Using plants deficient in both genes, *NBS1* and *TERT*, we found that plants exhibited severe genomic instability even in early generations. These phenotypes worsened with increasing generations on self-pollination and plants developed serious developmental defects leading to sterility in the 6^th^ generation. By comparing the length of telomeres in double and single mutants we observed accelerated and more frequent telomere shortening in the former.

## Results

### *tert/nbs1* mutants displayed severe morphologic developmental defects over increasing generations

**Figure 1:**
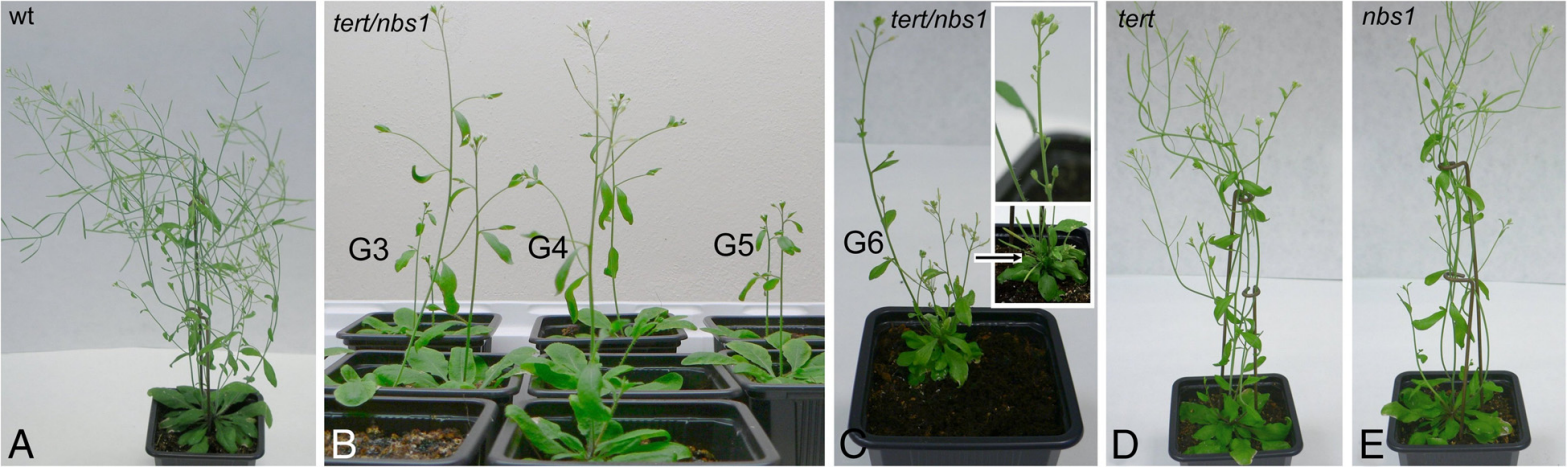
**Morphology of mutant plants.***nbs1* and *tert* homozygotes were crossed and from segregating F2 seedlings double homozygotes *tert/nbs1* were selected along with wild type plants (wt), *TERT/nbs1*, and *tert/NBS1* mutants. (**A**) G6 wild type plant, (**B**) double homozygous *tert/nbs1* mutants from G3, G4, and G5, line K12. (**C**) G6 homozygote *tert/nbs1*, Line L2. Inset - dwarf phenotypes of double mutant mutants, generation G6, Line K12. Note that the lower plant produced siliques with infertile seeds on a very short stem (arrow). (**D**) *tert* plant, line 69–2, G6. (**E**) *nbs1* plant, G6. All images represent 3 to 4 week old plants.

**Figure 2:**
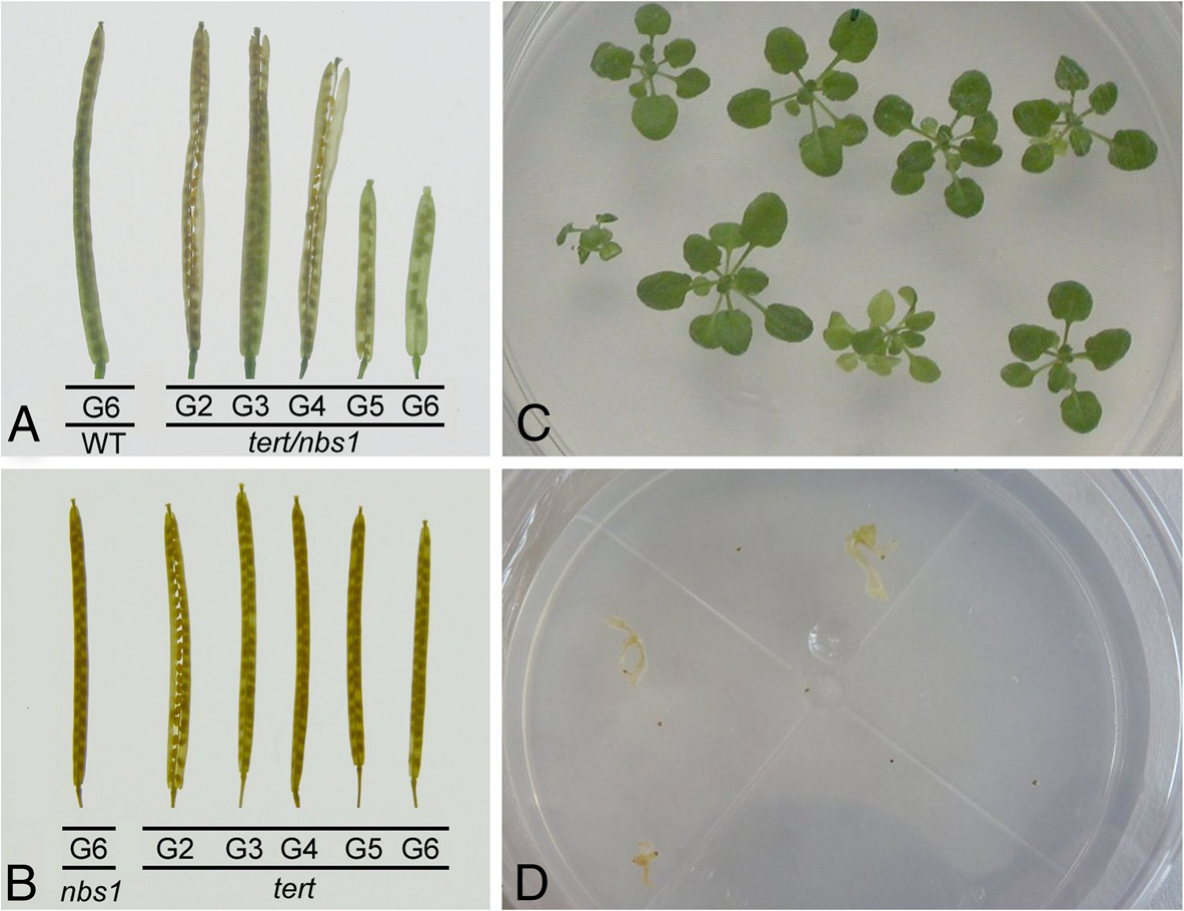
**Seed production and germination of late generation double mutants.** (**A**) Comparison of siliques from wt (G6) and G2 to G6 double homozygotes *tert/nbs1,* Line K12. Seeds from G5 plants were 45 % sterile in both lines, K12 and L2. G6 seeds were completely sterile. (**B**) *nbs1* siliques (G6) compared to *tert*, line 69–2 (G2 to G6). The fertility of *nbs1* and *tert* seeds was almost 100 %, irrespective of seed generation. (**C**-**D**) Seedlings of G7 plants on agar MS cultures, line K12. (**C**) Heterozygote *TERT/nbs1*. (**D**) *tert/nbs1* genotype seedlings did not germinate.

We performed a crossing between *nbs1* and *tert* homozygous plants. From segregating F2 seedlings, wild-type plants, *nbs1* mutants, two independent *tert* lines 69–1 and 69–2, as well as double homozygotes were selected by genotyping using T-DNA-specific and gene-specific primers. Two independent *tert/nbs1* lines were selected for onward analyses, K12 and L2. These lines are further referred to as generation 1 (G1). Upon self pollination, we obtained five consecutive generations, G2 to G6, until plants displayed defective morphology (Figure 1B), produced infertile seeds (Figure 2A), and were incapable of subsequent cultivation. Siliques from G5 plants, both lines, K12 and L2, contained only 12 ± 4 seeds with a 45% fertility. Although the number of seed pods from G6 plants was the same as in G5 lines, seeds failed to germinate in either soil or MS agar cultures (Figure 2D). By contrast, single *tert* mutant seeds exhibited no signs of fitness derogation from G2 to G6 in either line 69–1 or 69–2 (Figure 2B).

### Genomic instability of *tert/nbs1* is accelerated in comparison to single *tert* mutants

**Figure 3:**
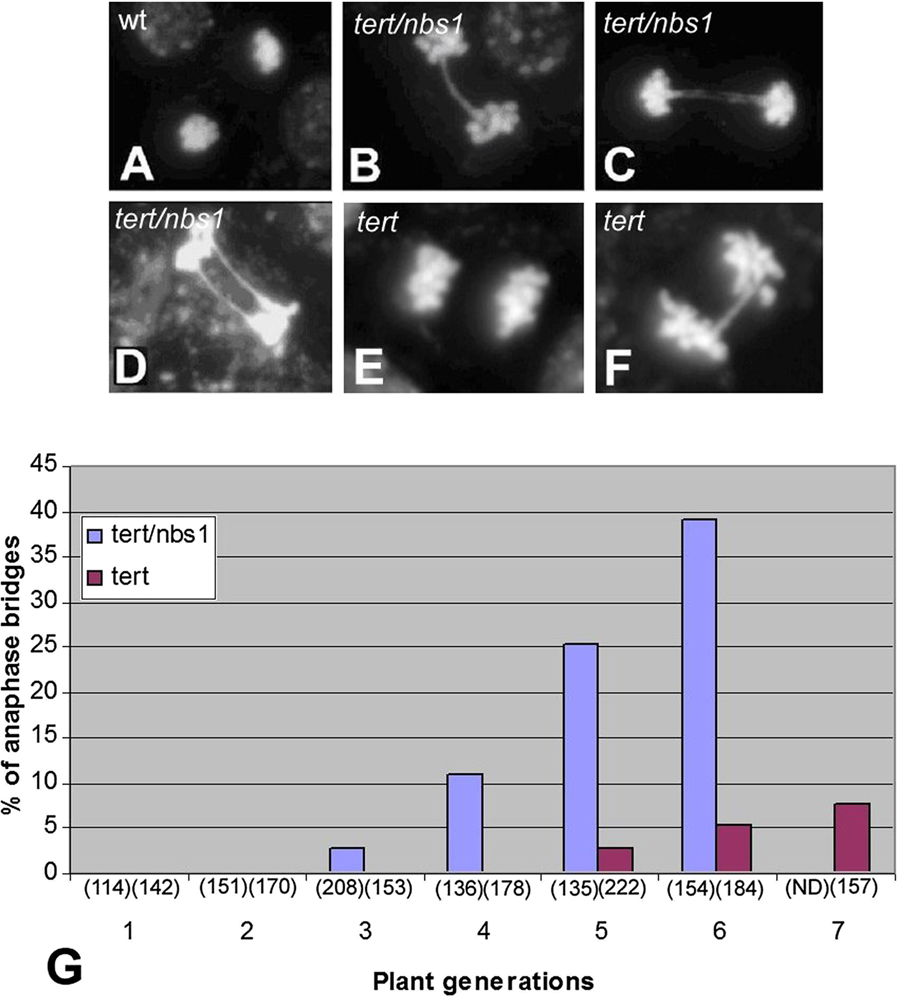
**Genomic instability of*tert/nbs1*manifested as mitotic anaphase bridges.** Pistils of experimental plants were treated for microscopy, tissues fixed, squashed and stained with 4',6-diamidino-2-phenylindole (DAPI). (**A**) Anaphase from G6 wild type plant, (**BD**) anaphases from double homozygous *tert/nbs1*, line K12, generation G6 possessing single anaphase bridge, twin bridge and two single bridges, respectively. (**EF**) Anaphases from single homozygous *tert* plants, line 69–2, generation G6. Whereas in this *tert* seed generation, the majority of anaphases exhibited no bridge (**E**), bridged anaphases were detected in 5.4% (**F**). (**G**) The frequency of bridges containing anaphases regardless of number of bridges between the respective anaphases are shown for *tert/nbs1*, line L2, (blue columns) and *tert*, line 69–2, (purple columns) in consecutive generations. The numbers of total anaphases scored are indicated in parentheses. No bridged anaphases were detected in *nbs1* heterozygotes or wild-type plants. The 95% Confidence Intervals [25] show significant differences between the values for generations G3 to G6. (ND = due to severe developmental defects, no plants were available for cytology).

We predicted accelerated signs of genome instability in somatic tissues of double homozygotes in comparison with single *tert* mutants. *Arabidopsis tert* mutants are known to be gradually more and more prone to chromosome fusions due to telomere de-protection in successive generations [24] with massive onset of fused chromosomes from the 6^th^ generation on. In our experiments, fused chromosomes were easily detected as anaphase bridges (Figure 3B–D). We used statistics [25] to compare the frequencies of anaphase bridges. Consistent with findings [24,26,27] single *tert* mutants appeared to possess 5.4% of anaphase bridges (Figure 3F) in G6 and increased incidence of bridges in G7 to 7.6% (Figure 3G). In comparison, double mutants *tert/nbs1* started to develop mitotic anaphase bridges from the G3, reaching 39.4 and 38.1% in lines K12 and L2 respectively, in generations G6 (Table 1). Microscopic inspection disclosed no anaphase bridges in *nbs1* generations, as in wild-type plants (*TERT/NBS1*) selected during the genotyping process. 

**Table 1:** **The frequency of anaphase bridges in *tert/nbs1 Arabidopsis *mutants during cultivation**

**Generation**	**Line**	**Anaphases scored**	**No of anaphase bridges**				**% total bridged anaphases**
			**Single**	**Double**	**Triple**	**Quadruple**	
G1	K12	100					0.0
G1	L2	131					0.0
G2	K12	112					0.0
G2	L2	91					0.0
G3	K12	95	4				4.2
G3	L2	357	6	3			2.5
G4	K12	248	25	4			11.7
G4	L2	42	3				7.1
G5	K12	348	57	28	1		22.1
G5	L2	331	53	32			25.7
G6	K12	614	130	101	10	2	39.4
G6	L2	381	67	59	16	3	38.1

In G1 to G2 no anaphase bridges were detected. Two independent lines of *tert/nbs1* double mutants were scored, K12 and L2. The 95 % Confidence Intervals [25] show that no statistical differences exist between the values for lines K12 and L2 in generations G3 to G6.

### Meiotic defects in *tert/nbs1* mutants

**Figure 4:**
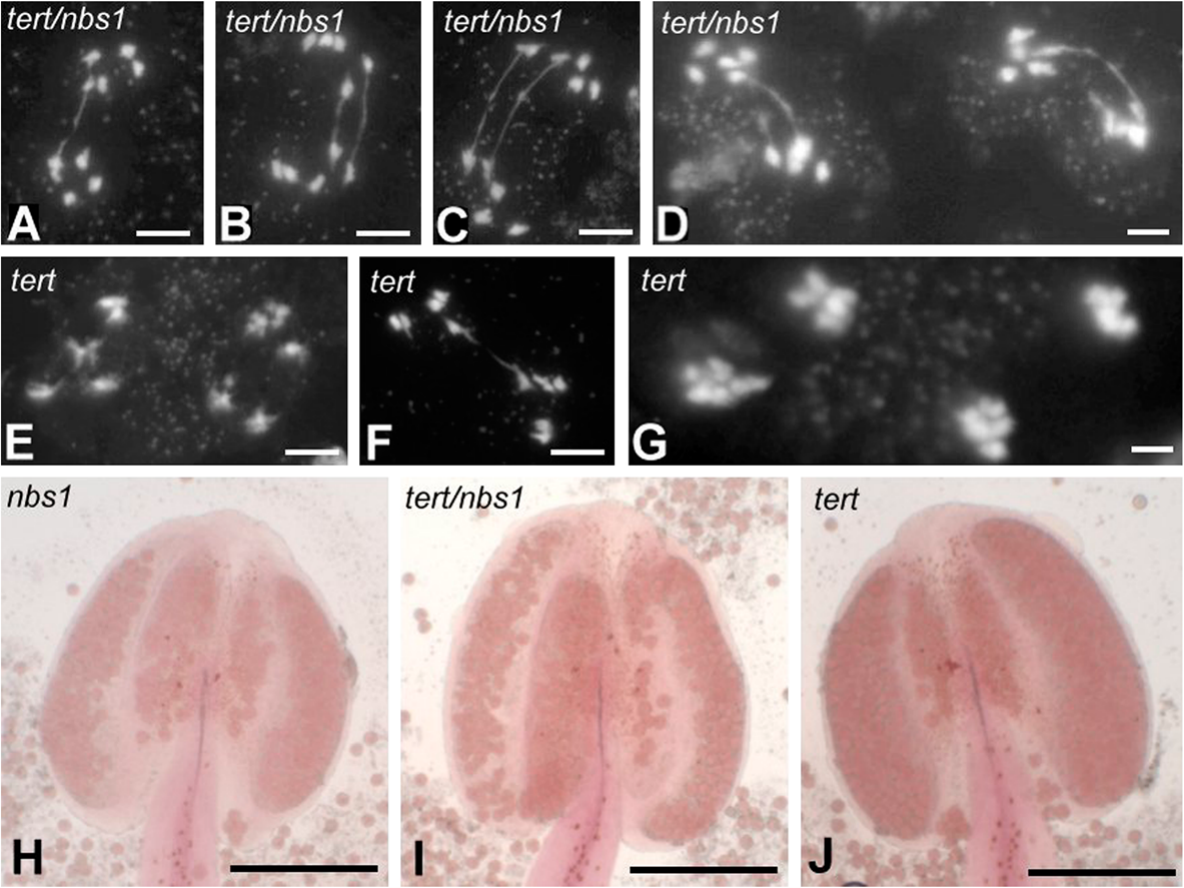
**Defects in male meiosis.** (**A**-**C**) Meiotic anaphases I in double homozygous *tert/nbs1* pollen mother cells, line K12, G2 generation. Bivalents are connected with one (**A**) or two bridges (**B**, **C**). (**D**) During anaphase II, segregating univalents are interconnected by bridges as a consequence of chromatid fusion in earlier phases of meiosis. Note the coequal pattern of dividing univalents. (**E**-**G**) *tert* mutants, line 69–2, G6. (**E**) In the majority of anaphase I preparations, no bridges were presented. (**F**) A bridge detected in anaphase 1 of *tert*, line 69–2, G6. (**G**) *tert* products of anaphase II are morphologically normal univalents (line 69–2, G6). (**H**-**J**) Anthers from G6 plants stained with aceto-carmine. (**H**) *nbs1*, (**I**) *tert/nbs1*, and (**J**) *tert*. Bars indicate 10 μm for (**A**-**G**) and 200 μm for (**H**-**J**).

Although microscopic inspection revealed no aberrations in male meiocytes from wild-type plants and *nbs1* mutants, double mutants (*tert/nbs1*) displayed numerous chromosome bridges between bivalents in anaphase I and between univalents in anaphase II (Figure 4A–D). These bridges were observable in preparations from pollen mother cells from G2 on. No other signs of meiotic aberrations were observed. Pachytene bivalents were fully aligned and male meiosis ended by morphologic perfect tetrads. Pollen viability of *tert/nbs1* was 100% (Figure 4I). From the analysis of *tert* mutants from G6, among 55 individual anaphase I figures scored, both lines 69–1 and 69–2, only two contained bridged bivalents (Figure 4F). Products of male meiosis were apparently normal for *tert* G6 plants (Figure 4G). Comparing aceto-carmine stained pollen grains in anthers, no differences between genotypes tested were noted and all pollen grains were apparently fertile (Figure 4H–J).

### Chromosomal fusion points of mutant cells exclusively involve chromosome termini

**Figure 5:**
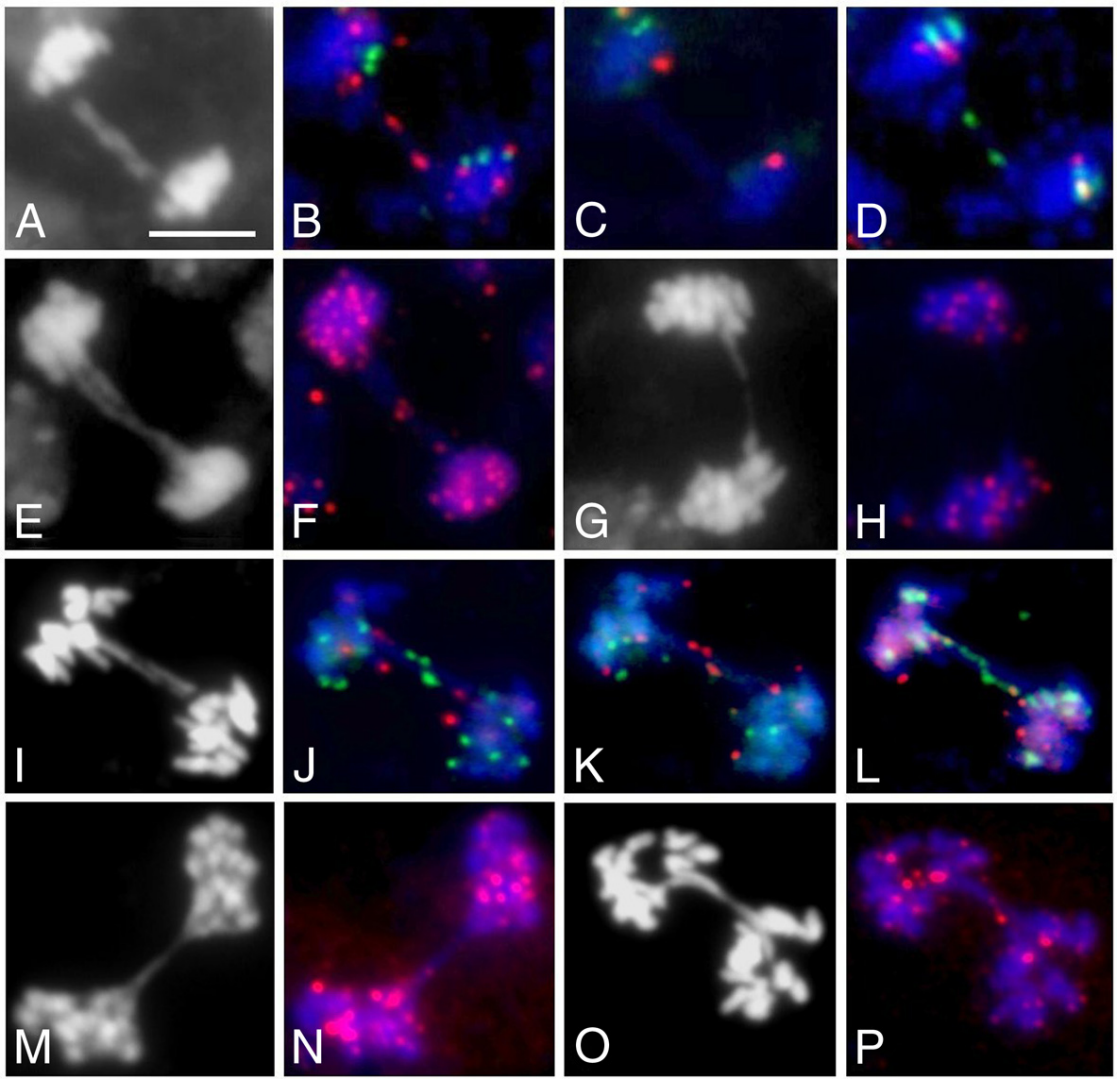
**Chromosome fusion points as revealed by FISH.** (**A**-**H**) *tert/nbs1* double homozygotes, (**I**-**P**) *tert*. (A-D) *tert/nbs1* mutant, generation G6, line K12. Sequential FISH on mitotic anaphase bridge revealed involvement of the right ends from chromosome 1. (**A**) DAPI stained anaphase with the bridge consisting of two chromatids. (**B**) The signals from BAC probe F5I6 (Cy3 labelled, red) are presented on both bridges. In the second FISH (**C**) no signals on the bridges are presented whereas in (**D**) the green probe (SpectrumGreen labelled, BAC F5I6) highlights the same positions as in (**B**). According to the FISH setup, alternation of colours unambiguously disclosed the fusion of the 1R chromosome end. (**E**-**H**) FISH with telomere-specific probe. Using telomere-specific Cy3-labelled PNA-C_3_TA_3_-probe, fusion points were detected on two chromatid anaphase bridges from line K12, generation G3 plants (**F**), whereas in higher generations (G4) the signals from PNA-probe were not detectable on the anaphase bridges (**H**). To demonstrate the morphology of bridges DAPI stained images are presented (**E**, **G**). (**I**) DAPI stained anaphase displaying twin anaphase bridges of *tert* mutant, line 69–2, generation G6. (**J**-**L**) Sequential FISH. (**J**, **K**) Left ends of chromosomes 3 were sequentially labelled by 3 L-specific T4P13 BAC, green in first FISH (**J**), and red in second FISH (**K**). In the third FISH, green signals from the probe for 45S rDNA illuminated the 2 L chromosome end (**L**). Thus, direct reciprocal fusions of 3 L and 2 L chromosome ends were proven. (**M**-**P**) Anaphase bridges from *tert* mutants probed by telomere PNA probe. (**N**) *tert* line 69–1, G5 and (**P**) line 69–2, generation G6. In both lines fusion points were decorated by telomere-specific probes from the generation G5 on. (**M**, **O**) DAPI stained anaphases with bridges. Bar represents 10 μm for all images.

In order to judge whether terminal or intercalary sites of chromosomes are involved in fusion sites of *tert/nbs1* mutants, we performed repeated rounds of bicolour FISH with chromosome-specific BAC probes directly adjacent to *Arabidopsis* telomeres as in [26]. On anaphase bridges of *tert/nbs1* mutants, we almost exclusively found signals from chromosome termini (Figure 5B–D). Using FISH with a directly labelled PNA telomere probe, we detected telomere tracts on a portion of bridges in G3 in both lines (Table 2), K12 (Figure 5F) and L2. From generation G4 on, telomere signals were not detected on the anaphase bridges of the double mutants (Figure 5H). This finding can be explained by the shortening of the telomere to an extent not detectable by the in situ hybridization procedure, consistent with our assumption that telomeres of the *tert/nbs1* mutants shorten considerably faster than these of plants with the knockout telomerase gene only. The anaphase bridges of the *tert* mutants (at least for generation G6) also contained FISH signals from telomere-adjacent BAC probes or chromosome terminal sequences as rDNAs (Figure 5J–L). As a rule, fused *tert* chromosomes contained telomere-specific signals in generations G5 and G6 (Table 2, Figure 5N and P). 

**Table 2:** **Comparison of telomeric signals on anaphase bridges of *tert/nbs1 *and *tert *mitoses using FISH with telomere-specific PNA probe**

***tert/nbs1***				***tert***			
**Generation**	**Line**	**Bridges**	**Telomeric signals**	**Generation**	**Line**	**Bridges**	**Telomeric signals**
G3	K12	4	4	G3	69-1	0	ND
G3	L2	9	7	G3	69-2	0	ND
G4	K12	29	0	G4	69-1	0	ND
G4	L2	0	ND	G4	69-2	0	ND
G5	K12	45	0	G5	69-1	4	4
G5	L2	65	0	G5	69-2	3	3
G6	K12	80	0	G6	69-1	10	10
G6	L2	47	0	G6	69-2	12	10

K12 and L2 represent independent lines of *tert/nbs1* mutants, whereas 69–1 and 69–2 are two independent *tert* mutants. ND applies to when no bridged anaphases were available and no FISH was performed. In *tert/nbs1* anaphase bridges were detected from G3 on, in both lines; in the majority of fused anaphase bridges telomere signals were detected. Nevertheless, from generation G4, telomeric signals were no longer detected. Anaphase bridges in *ter*t mutants started to occur from G5 in both lines, and in a prevailing manner the bridges bore telomere-specific signals.

**Figure 6:**
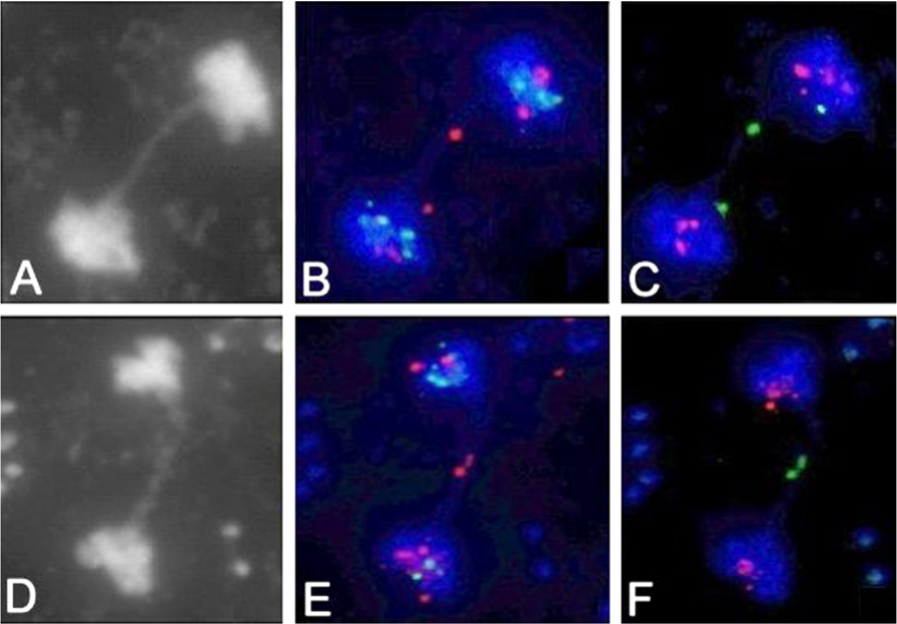
**Biased chromosome fusions.** (**A**-**F**) Anaphase bridges represent the involvement of the right arm of chromosome 2. Sequential FISH with telomere-adjacent 2R specific BAC clone revealed the fusion of 2R chromosome ends. The morphology of bridges is apparent from DAPI stained images (**A**, **D**). Using consecutively BAC F11L15 labelled with Cy3 (red, B and E) in the first FISH and labelled with SpectrumGreen (green, **C** and **F**), the direct contact of chromosome termini is highlighted. This respective alternation of signals during sequential FISH procedures was detected in the great majority of anaphase bridges tested in line K12. (**A**-**C**) Generation G3, (**D**-**F**) Generation G6.

From examination of the possible participation of respective chromosome ends forming anaphase bridges in *tert/nbs1* double mutants, we found a notable bias to fusions of the chromosome 2 right arm (Figure 6, Table 3). This more frequent involvement of 2R chromosome ends in fusions, pertaining exclusively to mutant line K12, was observable from G3 throughout G6. In *tert* mutants, both lines, 69–1 and 69–2, and *tert/nbs1* of the L2 line anaphase bridges basically carried random terminal signals.

**Table 3:** **Fusions of the right arms of chromosome 2 of *tert/nbs1 *and *tert *mutant *Arabidopsis *plants as revealed by FISH**

***tert/nbs1***					***tert***				
**Gen.**	**Line**	**Anaphase bridges tested by FISH**	**2R signals**	**2R signals (%)**	**Gen.**	**Line**	**Anaphase bridges tested by FISH**	**2R signals**	**2R signals (%)**
G3	K12	36	26	72.2	G3	69-1	0	ND	ND
G3	L2	41	0	0.0	G3	69-2	0	ND	ND
G4	K12	40	18	45.0	G4	69-1	0	ND	ND
G4	L2	45	3	6.7	G4	69-2	0	ND	ND
G5	K12	32	27	84.4	G5	69-1	6	0	0.0
G5	L2	37	0	0.0	G5	69-2	5	0	0.0
G6	K12	42	33	78.6	G6	69-1	10	1	10.0
G6	L2	54	2	3.7	G6	69-2	12	0	0.0

Three sequential FISH with chromosome-end specific probes revealed enhanced occurrence of 2R chromosome ends in line K12 of the *tert/nbs1* double mutants. In the sibling line K2, the fusion points were represented by random chromosome-arm interactions. In *tert* mutants lines 69–1 and 69–2, the occurrence of anaphase bridges starts from G5 on. Although the number of anaphase bridges available for analyses was rather low, cf. Figure 3G, only in one case the 2R-2R fusion had been detected. ND - no anaphases available.

### Telomeres of *tert/nbs1* plants rapidly shorten and to a greater extent than the *tert* lines

**Figure 7:**
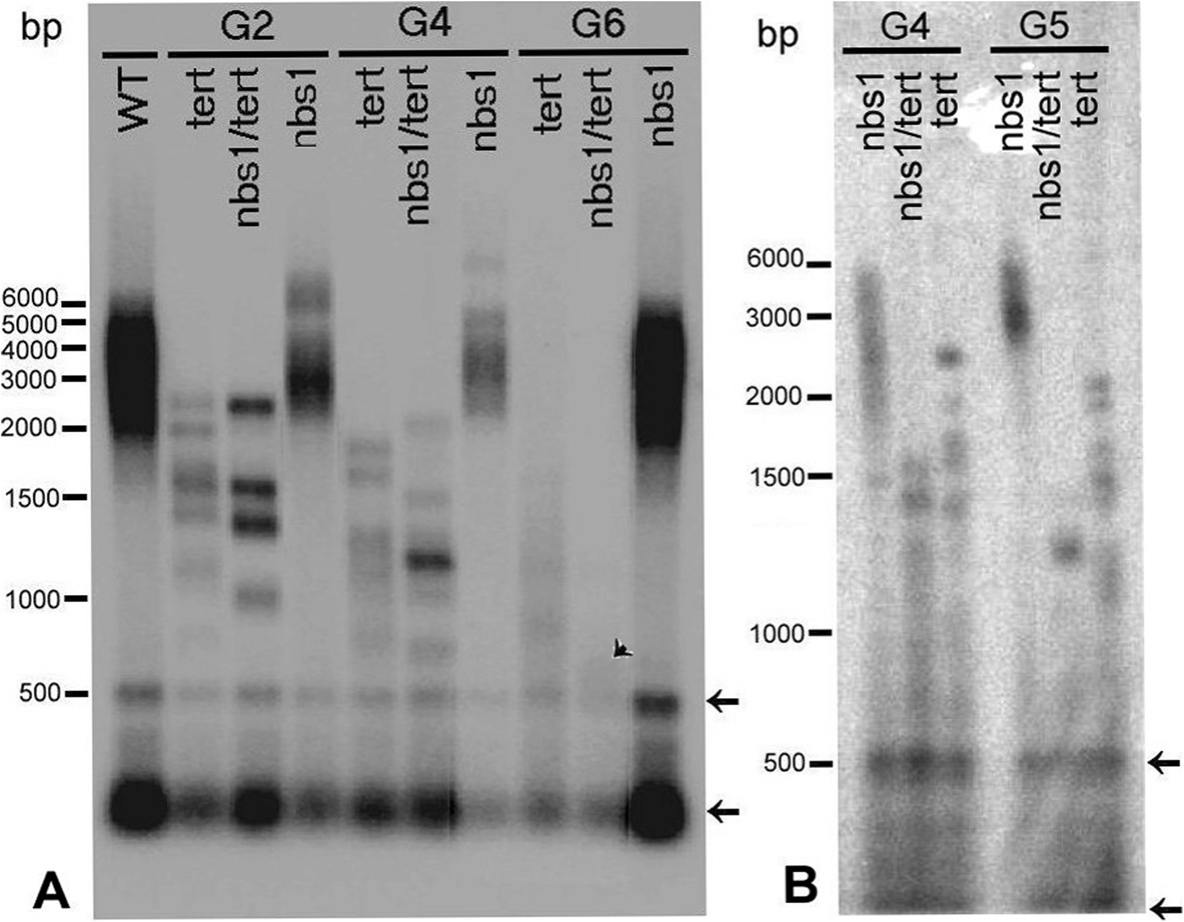
**TRF analysis of mutant plants.** Terminal telomere fragments were obtained by digestion of DNA isolated from representative plants in second, fourth, and terminal generations (G2, G4, G6) by *Tru1*I restriction endonuclease, separated on 1% agarose gel, Southern transferred, and visualized by ^32^P labelled C_3_TA_3_ probe. (**A**) Wild type and *nbs1* mutants had telomere lengths 5000 to 2000 bp. Gradual shortening of *tert* mutants (line 69–2) is accompanied by more dramatic telomere shortening in double homozygotes *tert/nbs1*, (line K12) to as short as 600 bp in G6 (arrowhead). The differences in the intensity of signals reflect slight variations in the amount of DNA loaded in each lane. (**B**) The comparison of TRF fragments of plants G4 and G5. Gradual decline in the length of the fragments of double homozygotes *tert/nbs1* (line L2) from 1600–700 bp to 1300–700 bp is evident between consecutive generations. The ranges of the lengths are markedly shorter than for *tert* (line 69–1) displaying 2500–1400 bp and 2000–1000 bp respectively for generations G4 and G5. Arrows point to the interstitial telomere sequences in (**A**) and (**B**).

To assess the overall lengths of telomere tracts in somatic cells of the *tert/nbs1* mutants, we applied the TRF method (terminal restriction fragment analysis) to DNAs isolated from individual plants of various generations and from both lines, K12 and L2. Whereas wild-type *Arabidopsis thaliana* of the Columbia ecotype possess telomeres with lengths ranging from 2 to 5 kb, mutant lines displayed remarkable shortening. When comparing *tert* and *tert/nbs1,* the telomeres of the latter shortened markedly faster (Figure 7). In contrast to the wild-type, we found in both mutants banded patterns where individual bands represent individual telomere lengths ranging from 4 to 1 kb for both *tert* and *tert/nbs1* in G1. With successive generations of plants, the length differences were more apparent, reaching 1.5 to 0.7 kb for *tert* of G6 plants, line 69–2, and as little as 0.6 kb for *tert/nbs1* G6 plants (Figure 7A, arrowhead). The trend of the accelerated telomere shortening of *tert/nbs1* double mutants is also visible when comparing G4 to G5 plants (Figure 7B). Homozygous *nbs1* plants displayed no marks of telomere shortening in the same experiments.

## Discussion

### Experimental setup and plant morphology

Individual constituents of the MRN plant complex have been stepwise analyzed in recent years. *MRE11* T-DNA insertion mutants have been described as having severe impact on plant growth and the fertility of homozygotes [20,28]. Similarly, mutant *Arabidopsis* plants with T-DNA within a *RAD50* gene were sterile [18,29,30] and capable of survival only as in vitro cultures.

In this study, we analyzed the possible role of NBS1 in telomere length homeostasis. We used the same *Arabidopsis* mutant line GK-570B09 as in [23]. T-DNA insertion resulted in N-terminal truncated protein lacking functional FHA and BRCT domains, whereas the MRE11 binding domain was retained. This deficiency did not affect the growth or fertility of the *nbs1/nbs1* homozygotes (Figure 1), as in [23]. Homozygous plants deficient in *TERT* gene have been extensively studied [24,26,31,32] and shown to possess no telomerase activity [31]. Nevertheless, the plants were viable in our experiments and provided fertile seeds over seven generations, similar to [31]. By contrast, double homozygous *tert/nbs1* plants exhibited signs of developmental impairment from the G3 generation (Figure 1B), accompanied by gradual decline in seed production (Figure 2A). Although the dwarf G6 plants provided seeds, they had no germination capacity (Figure 2D).

### Genomic instability of the *tert/nbs1* homozygotes

The measure of genomic instability in our experiments was the occurrence of mitotic anaphase bridges (Figure 3B–D). Whereas in wild-type plants, anaphase bridges were not detected, yet in double homozygotes *tert/nbs1* the presence of anaphase bridges was detected from G3 on (Figure 3G). Over the next three seed generations (G6 was terminal), the percentage of bridged anaphases increased dramatically, reaching almost 40% in G6 (Figure 3). Comparing the two independent *tert/nbs1* lines, K12 and L2, no difference in frequency of bridges was found (Table 1). In contrast, *tert* mutants began to exhibit anaphase bridges in G5, but the proportion remained relatively low: 2.7%, 5.4%, and 7.6% for G5, G6, and G7 (Figure 3G). Since *nbs1* homozygotes did not display any anaphase bridge under microscopic evaluation, markedly enhanced frequency and accelerated occurrence of anaphase bridges observed in our double homozygotes can only be explained by the synergistic interaction of both deficiencies.

We also detected anaphase bridges in meiotic anaphases of pollen mother cells of the double mutants (Figure 4) while other stages of meiosis were apparently normal. We observed no defective meiosis of single *nbs1* mutants across any generations studied. Employing this identical *nbs1* mutant but in an *atm* background, massive defects in early stages of meiosis occurred, implying a role for NBS1 in the control of meiotic progression which was independent of ATM signaling [23].

### Fused chromosomes of *tert/nbs1* entirely involved chromosomal termini

The fusion of chromosomes by the NHEJ mechanism is a consequence of telomere uncapping and can be the result of random fusions of various replicated chromosomes or the consequence of the fusion of sister chromatids of the same chromosome [27]. FISH analysis with BAC probes originating from chromosomal sites directly adjacent to telomeres of *Arabidopsis* chromosomes revealed that the majority of bridges contained terminal BAC signals (Figure 5B–D) and, specifically, no combination of signals from various chromosomes. Therefore the prevalent mechanism of chromosomal joining was the fusion of sister chromatids. This is in contrast to *tert* mutants, where various chromosome termini were randomly recruited to fusions in both lines, 69–1 and 69–2 (Figure 5J–L). In the sparsely occurring bridges of G3 of double homozygotes, we were able to observe clear telomere signals on the bridges in a high proportion (Figure 5F, Table 2) which was in striking contrast to higher generations (G4 to G6) where telomere tracts on the anaphase bridges were never detected (Figure 5H). In contrast, *tert* mutants regularly contained traces of telomere sequences, at least in G5 and G6 (Figure 5N and P; Table 2). The loss of telomere DNA has been noticed on the mitotic bridges of the *Arabidopsis rad50* mutant [19]. However, double hetrozygotes *tert/rad50* frequently contained the telomere repeats [19], thus arguing for two different roles of the RAD50 in either the de-protection of telomeres or with telomeres already shortened by the absence of TERT.

In a number of *Arabidopsis* mutants exhibiting somatic anaphase bridges the participation of either the homologous chromosomes or one-chromatid fusions has frequently been described; this holds for *mre11-3/ku70* mutant (44% of FISH-identified fusions were decorated by a single telomere-adjacent probe) [20], or for *atm/tert* mutants where a single chromosome end at fusion junctions was also detected [33]. Similarly, when we tested the feasibility of a sequential FISH assay for the assessment of chromosome-end fusions [26] by analyzing G5 *tert* plants, we found that 47% of all identified fusions contained the signal from the same chromosome end. In the present study we describe the prevalent occurrence of the fusion of 2R chromosome ends in the double mutant, line K12 (Figure 6, Table 3). This can be explained by critically shortened 2R-telomere in early generation and by clonal propagation of this impairment through somatic tissues and germlines for succeeding generations. Albeit that the cause of the chromosome fusions in these various genetic backgrounds was rather different, a substantial proportion of the fusions of uncapped telomeres were accounted for by sister chromatid interactions.

### A possible role of NBS1 in the protection of telomeres

We observed an increasing occurrence and higher frequency of anaphase bridges in double homozygotes than in wild-type or single *tert* mutants. From measuring the overall telomere length using terminal restriction fragment (TRF) analysis, we uncovered faster shortening of telomeres from individual chromosomes of the double mutants with respect to *tert* mutants (Figure 7). At the same time, the length of telomeres in *nbs1* was unaffected and comparable with that of wild-type plants.

Substantial progress in the knowledge of RAD50 and MRE11 and their role in the DNA repair and telomere biology of *Arabidopsis* has been made while NBS1-related data remain to be collected. From our experiments, we cannot gauge whether the synergism observed is due to a functional shortcoming or existence of a direct physical interaction of NBS1 with telomeres in plants. Possibly the first direct observation of the co-localization of the NBS1 protein with telomeres (TRF2 proteins) was found in HeLa cells during the S phase of the cell cycle, suggesting a possible role in telomere replication [14]. In the same year, interaction of NBS1 with TRF1 proteins was also observed in immortalized telomerase negative cells [13]. In experiments with cultured cells isolated from patients with the NBS1 syndrome, protein NBS1 was implicated as a positive regulator of the telomerase but the question remains as to whether the protein recruits this enzyme to the telomeres [34]. Using the cre-lox recombination system for the inactivation of TRF2 and NBS1 in mouse embryonal fibroblasts, the recruitment of NBS1 to dysfunctional telomeres was found to be cell cycle dependent [15]: the NHEJ was induced during G1 through ATM-dependent signalling, and, the NHEJ was repressed in G2 via a 5' end-resection mechanism which produced an NHEJ-incompatible 3' overhang on replicated chromosomes.

In the TRF analysis, there were observed two different patterns, smeared signals for both, wild-type and *nbs1* plants representing various telomere lengths, and a number of individual bands representing single telomeres for plants with shortened telomeres (Figure 7). These discrete bands are hallmarks of telomere shortening [31] and provide evidence that shortened telomeres are clonally propagated in plant tissues and/or consecutive generations. Gross developmental defects of *Arabidopsis* plants and abundant end-to-end chromosome fusions in *atm/tert* mutants prove that all cells in the plant embryo inherit a critically shortened telomere [33]. Although analyses showed that the short telomere arose as a consequence of a large telomere rapid deletion (TRD) event, FISH analysis revealed that fused chromosomes exhibited overrepresented single chromosome end. Notably, in our *tert/nbs1* mutants, line K12, we detected overrepresented 2R chromosome ends at the fusion points in all generations analyzed by FISH (Figure 6). Interestingly, telomere shortening was detected from generation G2 (Figure 7A), far earlier than end-to-end chromosome fusions were detected in G3. This fact strongly supports the existence of a synergism between NBS1 and telomerase metabolism in *Arabidopsis* and excludes the possibility of the consequent effect of *nbs1* mutation on de-protected telomeres.

We did not address the issue of whether the deficiency of NBS1 alone was responsible for the observed phenotypes in double mutants or whether there was a disturbance in the proper assembly of the MRN complex with subsequent impact on the phenotypes. Hypomorphic mutation in the *NBS1* gene in question, results in transcript truncated for the N-terminal portion thus lacking FHA and BRCT domains, whereas the C-terminal domain containing MRE11-binding and putative ATM-interaction motif sites are retained [23]. For this reason, the assembly of the MRN complex seems to be unaffected. The existence of phosphopeptide binding domains within the FHA and BRCT motifs in functional NBS1 renders multiple interactions with a plethora of proteins requiring phosphorylation to function in DNA-damage repair or telomere maintenance [35-37]. Thus, the dysfunction of the NBS1 can be entailed to the missing FHA/BRCT domain.

## Conclusions

Direct physical contact/presence of NBS1 on telomeres and their function in telomere maintenance has been substantiated in the case of humans but not in plants. Understanding here is hampered by the fact that no shelterin counterparts had been described in plants to date. With our research we now provide indirect evidence of mutual interactions between NBS1 and telomeres. It is still unclear at this time whether protein truncation at the N-terminal domain is responsible for the phenotypes observed in *tert/nbs1* mutants or if the disruption of certain unknown telomere-protecting pathways is responsible.

## Methods

### *Arabidopsis thaliana* mutants

Mutant plants (both Columbia background) were cultivated in an environmental growth chamber at 21°C under 16/8 h light/dark period. When cultured on agar plates, Murashige and Skoog medium including B5 vitamins (Duchefa) supplemented with 20 g/L of sucrose in 0.8% Plant Agar (Duchefa) was used and seeds germinated in permanent light. *tert* mutants were obtained from the laboratory of Dr. Karel Riha (described in [31]). *nbs1* mutant line 570B09 was obtained from GABI-KAT [38]. Homozygous *nbs1* and *tert* plants were selected by PCR genotyping using T-DNA-specific and gene-specific primers for *tert* mutation LB6: GAACATCGG TCTCAATGCAA [31] and TERT6: CTAGGACATATCCATCAAGGGCT [31] and for *nbs1* mutation NBSF: GGTTGTCCTTAATTCCGCTTG and 8409: ATATTGACCATCATACTCATTGC. Homozygotes were crossed and from segregating F2 seedlings double homozygotes *tert/nbs1*, as well as wild-type, *tert* (two independent lines, 69–1 and 69–2), and *nbs1* single mutants were selected and affirmed by PCR using TERT7: GAAAGGAAGCTGTATTGCACGAA [31], TERT6 and NBSF and NBFR: GGCTGTATCCAGGAATTTCG, respectively. From double homozygotes, two independent lines were selected for further analyses, K12 and L2. These are further referred to as generation 1 (G1). With self pollination we obtained five consecutive generations, G2 to G6 until plants produced infertile seeds.

### DNA extraction and Terminal Restriction Fragment (TRF) analysis

DNA was isolated according to [39] from inflorescences or young leaves 3 weeks after germination. TRF analysis was performed according to [40]. Briefly, DNAs were digested with *Tru1*I (Promega) overnight at 65°C and electrophoresed in 1% agarose. After Southern blotting, the membranes were hybridized with 32P-gamma-ATP end labelled (T3AG3)4 oligonucleotide. Radioactive signals were visualized on Typhoon FLA 9500 scanner and analyzed using ImageQuant software (GE Healthcare).

### Preparation of mitotic and meiotic chromosomes

For the preparation of mitotic samples, whole terminal inflorescences were fixed in 3 : 1 mixture of ethanol and acetic acid. The inflorescences were washed with tap water and transferred to 10 mM citrate buffer (pH 4.5). Pistils were excised from floral buds under dissection microscope and macerated using a mixture of 0.5% Onozuka cellulase (SERVA Electrophoresis Ltd., Heidelberg, Germany) and 0.5% Pectolyase (Sigma Chemical Co., Saint Louis, MO, USA) at 37°C in a moist chamber for 3 h. Pistils were transferred into a drop of 60% acetic acid on slides and squashed under a cover-slip. After freezing of slides in liquid nitrogen and cover-slips removal, the slides were postfixed in 3:1 mixture of ethanol and acetic acid and air dried. Meiotic preparations were obtained after the same maceration procedure from anthers of immature flower buds (1–2 mm long).

### Anthers staining

Anthers were fixed/stained in 1% solution of carmine in 45% acetic acid for 30 min and then gently squashed under a cover-slip. Mature and healthy pollen grains stain in light purple.

### Bicolour FISH and labelling of probes

As FISH probes for individual termini of *Arabidopsis* chromosomes, bacterial artificial chromosomes (BAC) clones were selected and using repeated FISH procedures the fusion points were assessed exactly as in [32]. BAC clones for the identification of each particular Arabidopsis chromosome end were F6F3, F5I6, F11L15, T4P13, F16M2, F6N15, T5J17, F7J8, and K9I9. These BACs were obtained from the Arabidopsis Biological Resource Center, Columbus, Ohio, USA. BAC DNAs were isolated from bacterial cultures using QIAGEN Plasmid Midi Kit (QIAGEN Inc., Valencia, CA, USA) exactly according to manufacturer recommendations. The probe for identifying the left arms of chromosomes 2 and 4 was the internal 2478 bp (*Eco*R I) fragment of the 25S-rRNA gene [41]. Probes were labelled either with SpectrumGreen-dUTP (Abbott Molecular, Illinois, USA) or Cy3-dUTP (GE Healthcare UK Ltd., Little Chalfont, England) using Nick Translation Mix (Roche Applied Science, Mannheim, Germany). Telomeric tracts were visualised by custom synthesized Cy3-tagged PNA probe CCCTAAACCCTAAA (Applied Biosystems, Bedford, MA, USA).

### FISH procedures and acquisition of images

Microscope preparations were digested with 100 μg/ml in 2xSSC RNase A (Promega, Madison, WI, USA), for 1 h at 37°C and pepsin (Sigma, 50 μg/ml in 0.01 N HCl, 10 min, RT). Slides were post-fixed in 3.7% neutral formaldehyde. The hybridization mix contained 50 ng of each labelled BAC DNA and/or of 20 ng of labelled rDNA probe, 10% dextran sulphate, and 50% formamide in 2xSSC. Heat-denatured hybridization mixture was applied to slides, the slides were covered with cover-slips, and subjected to heat denaturation. After hybridization at 37°C overnight, the slides were stringently washed in 0.1x SSC at 42°C. Preparations were mounted in Vectashield (Vector Laboratories, Peterborough, UK) with 1 μg/ml 4',6-diamidino-2-phenylindole (DAPI, Sigma). For telomere PNA probe, the stringency of the hybridization was increased using 60% formamide in the probe mix. Microscope images were visualized using the Olympus AX 70 fluorescent microscope (Olympus, Tokyo, Japan) equipped with AxioCam MRm (Carl Zeiss Inc., Goettingen, Germany) camera and processed with ISIS imaging software (MetaSystems Ltd., Altlussheim, Germany).

## Abbreviations

ATM: Ataxia telangiectasia mutated; ATR: Ataxia telangiectasia and Rad 3 related; BAC: Bacterial artificial chromosome; *BRCA1*: Breast cancer 1 gene; BRCT: BRCA1 C terminus domain; DSB: Double strand break; FHA: Forkhead-associated domain; KU70: KU70 protein; *MRE11*: Meiotic recombination 11 gene; MRN: MRE11-RAD50-NBS1 complex; *NBS1*: Nijmegen breakage syndrome 1 gene; NHEJ: Non-homologous-end-joining; PNA: Peptide nucleic acid; RAD50: DNA repair protein; *TERT*: Telomerase reverse transcriptase; TRD: Telomere rapid deletion; TRF: Terminal restriction fragment analysis; TRF1: Telomeric repeat binding factor 1; TRF2: Telomeric repeat binding factor 2; XRS2: X-rays sensitive, yeast DNA repair protein.

## Competing interests

The authors declare that they have no competing interests.

## Authors' contributions

JS conceived the concept, LN and JS performed the experiments, and JS and LN wrote the paper. Both authors read and approved the final manuscript.
